# How Does Adjacent Land Use Influence Sediment Metals Content and Potential Ecological Risk in the Hongze Lake Wetland?

**DOI:** 10.3390/ijerph191610079

**Published:** 2022-08-15

**Authors:** Yanhui Guo, Yongfeng Xu, Chenming Zhu, Pingping Li, Yongli Zhu, Jiangang Han

**Affiliations:** 1 College of Biology and the Environment, Nanjing Forestry University, Nanjing 210037, China; 2 National Positioning Observation Station of Hongze Lake Wetland Ecosystem in Jiangsu Province, Hongze, Huai’an 223100, China; 3 Collaborative Innovation Center of Sustainable Forestry in Southern China of Jiangsu Province, Nanjing Forestry University, Nanjing 210037, China

**Keywords:** metal risk assessment, lake wetland, sediment, land use

## Abstract

Metal pollution in lake wetlands has become increasingly serious in China and worldwide due to the rapid growth of urbanization and agricultural activities. However, comprehensive assessments of metal pollution in lake wetland sediments that are associated with land use change have been limited from an international perspective. Metal concentrations (As, Cd, Cr, Cu, Hg, Mn, Pb, and Zn) were measured in the surface soils and surrounding sediments of five land use types in the eastern Hongze Lake wetlands, including Farmland (FL), Culture Ponds (CP), Reed Land (RL), Poplar Forests (PF), and Willow Forests (WF). The metal pollution status was assessed using the geo-accumulation index and the potential ecological risk index; The results showed that the average concentrations of As, Cd, Mn, and Zn in the surface soils and As, Cd, Cu, and Zn in the sediments, exceeded the background values of Jiangsu Province, China. The FL soils and surrounding sediments were moderately contaminated with As, whereas the sediments surrounding the CP were uncontaminated to moderately contaminated with Cd. Metal pollution in both soils and sediments was greater on farmland than on other types of land use. Furthermore, there were significant positive correlations between the values of the soil risk index and the values of the surrounding sediment risk index. Correlation analysis (CA) and principal component analysis (PCA) found that metals may be derived from agricultural activities such as the application of chemical and organic fertilizers, as well as domestic sewage, industrial wastewater, and geological anomalies. These findings shed new light on the quantitative impacts of adjacent land use practices on sediment metal pollution and provide a scientific foundation for wetland management decision-making.

## 1. Introduction

Lake wetlands are aquatic-terrestrial ecotones with unique hydrological, biological, biophysical, and landscape features that can provide humans with valuable ecosystem services such as freshwater provision, water purification, flood attenuation, climatic regulation, habitat for wildlife, and recreation [1,2]. The lake-wetland ecosystem is one of the most important ecosystems on the planet [3], but it has been severely impacted by rapid urbanization and other land use changes in recent decades. According to Xu et al. [4], agricultural land and urban expansion are the major factors causing wetland loss in China. They accounted for 47.7% and 13.8% of the total area of the loss in China from 2000 to 2015.

As a land use alters to support human life, the conversion of lake wetlands to farmland, aquaculture ponds, and human settlements provides essential ecosystem goods at the expense of the deterioration of the wetland environment [5]. In China, pollution is the primary cause of wetland degradation [6]. This is because land use is closely linked to anthropogenic activities, which can cause pollutants to enter waterways. The hydrology and water quality of wetlands are directly threatened by wastewater discharges from industry, agriculture, animal husbandry, and aquaculture. Large amounts of pollutants (nutrients and metals) were discharged into wetlands, causing damage to the integrity of the structure and function of lake wetlands since the1980s in China [7].

The accumulation of metals in lake sediments has attracted increasing attention in the environmental research of lake wetlands because of their potential biological toxicity, environmental durability, and biological accumulation in recent years [8,9,10]. Lead (Pb), for example, is the most bioconcentrated element in certain aquatic organisms. It can harm the nervous and hematopoietic systems of humans, and cause irreversible harm to children’s psychological, intellectual, and behavioral development as well [11,12]. Mercury (Hg) has the potential to harm both the renal and nervous systems, while arsenic (As) and cadmium (Cd) have negative effects on the skin, blood vessels, nervous system, lung, kidney, prostate, and bone [13,14]. Metals have a long-term impact on aquatic ecosystems because they do not degrade in the environment [15]. Most metals are attached to fine-grained particulates after being introduced into the aquatic ecosystem and, as a result of settling, accumulate in the bottom sediments where they may cause adverse biological effects even if the water quality criteria are not exceeded [16]. Therefore, there is a strong need to investigate metal pollution in lake sediments and assess the potential ecological risks that are posed by metals in order to protect the corresponding aquatic ecological environment and human health. Current lake sediment pollution research focuses on metal pollution characteristics and associated risk assessment [10,17,18,19], metal enrichment status and history [20], and source identification of metals [21,22]. However, very limited research has been done to investigate the influence of adjacent land use practices on the sediment metal pollution in lake wetlands. In addition, the potential relationships between the metal pollution in the topsoil of adjacent land use and those in lake sediments are not well-understood.

Hongze Lake is the fourth largest freshwater lake in China and the first natural sink for transferring drinking water along the East Route of the South-to-North Water Diversion Project (SNWDP-ER), which has been developed to solve the problem of water shortages in North China [23]. As the intersection point of the mainstream and branches, the eastern Hongze Lake wetland is both a sink for pollutants from the upper reaches of the Huai River and a source of the lower reaches of Huai River and SNWDP-ER [24]. Therefore, the quality of soil and sediment in eastern Hongze Lake wetlands is critical, which may affect the Project’s success or failure. However, the land use patterns of this area have changed in the past decades under the pressure of local economic development [25]. Culture pond and farmland area showed an increasing trend while open water area and the community of arbor and shrub plants showed a decreasing trend [26]. Land use changes in this region have resulted in serious environmental issues, such as declining water quality and soil and sediment pollution, with metal pollution becoming a major issue as a result of intensive agriculture and aquaculture’s rapid development [27,28]. Previous research on the metal pollution in soils and sediments of Hongze Lake region focused on contaminant survey and risk assessment on a large scale or for a single land use type. Yu et al. [27] discovered that the concentrations of Cd, As, and copper (Cu) in the sediments of Hongze Lake significantly exceeded the Chinese soil quality standard’s grade II level. The presence of As and chromium (Cr) in the Hongze Lake sediments was linked to human activity in the basin, according to correlation analyses [29]. However, there are not enough quantitative and insight studies about the impact of different land use types in Hongze wetlands on metal contents in wetland sediments. In addition, metals would be continuously introduced into the environment and accumulated in soils and sediments due to ongoing and excessive anthropogenic activities in this region, putting the lake ecosystem and human health at risk. Therefore, the potential ecological risk of metal contamination needs to be thoroughly investigated.

Understanding the effects of land use practices in lake wetlands on metal pollution of soils and lake sediments is critical for improving lake ecological security and preserving wetland ecosystem services. Thus, the objectives of this study were to (1) investigate the impact of five different types of land use on the contents of As, Cd, Cr, Cu, Hg, Manganese (Mn), Pb, and Zinc (Zn) in surface soils and sediments; (2) assess the metal pollution status and its potential ecological risk in soils and sediments using a geo-accumulation index (*I_geo_*) and the potential ecological risk index (*RI*); and (3) identify the possible sources of these metals in soils and sediments in relation to five different types of land use. The findings from this study will support decision-makers in developing effective environmental monitoring and efficient management strategies to minimize the impacts of local pollutants.

## 2. Materials and Methods

### 2.1. Study Area

**Figure 1:**
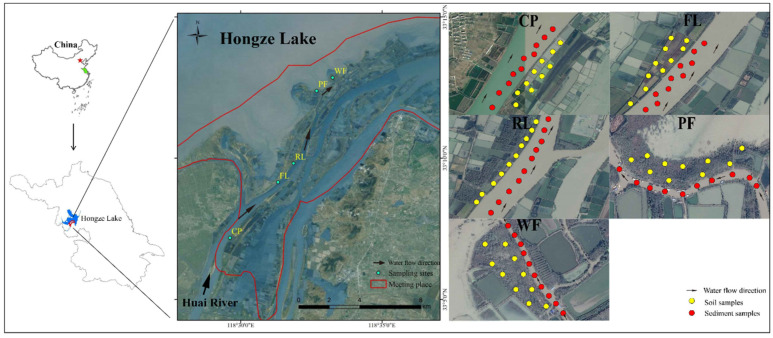
Location of the sampling sites for five types of land use (CP, FL, RL, PF, and WF) in this study. CP—Culture pond, FL—Farmland, RL—Reed land, PF—Poplar forest, and WF—Willow forest.

The study area is a wetland (33.166° N–33.188° N, 118.454° E–118.692° E) that is formed by the confluence of the Huai River and Hongze Lake in Jiangsu Province, China, with an area of approximately 14,700 hm^2^ (Figure 1). The investigation of metal contamination and the associated potential ecological risk on five typical land use types was undertaken in this study: culture pond (CP), farmland (FL), reed land (RL), poplar forest (PF), and willow forest (WF). Culture ponds are the most common land use type, accounting for about 16.28% of the total area and are primarily used for Chinese mitten crab intensive aquaculture. In the last two decades, farmland has sprung up in this area [26]. Before our study, the site had been cultivated under wheat-rice double cropping with chemical fertilizer inputs for 13 years. Besides, the poplar and willow forests are planted artificially with chemical fertilizers and herbicides that were used in the first two years. In contrast, the reed land is natural without significant human impact.

### 2.2. Sample Collection and Analysis

In June 2016, fifty surface soil (0–20 cm) samples were collected across the wetland area, with ten sampling sites for each land use type. A total of fifty sediment (0–20 cm) samples were collected from the lake sediment that was adjacent to the fifty surface soil samples for each type of land use (Figure 1). The soil samples are denoted by ‘so’ and the sediment samples ‘sd’ after each land use abbreviation, such as CP*_so_* and CP*_sd_*. Each surface soil or sediment sample was a composite sample that was bulked from three sub-samples that were taken around the collection point according to the principles of S shape with an inter-space of 10 m. All the soil and sediment samples were placed into polyethylene bags, returned to the laboratory, and air-dried at room temperature for four weeks. Stones and other debris were removed by sieving through a 2 mm nylon sieve. The samples were then ground with a pestle and mortar until all the particles passed a 0.15 mm nylon sieve for determining the soil and sediment chemical properties.

The concentrations of As, Cd, Cr, Cu, Hg, Mn, Pb, and Zn were determined for all the soil and sediment samples. For As and Hg analysis, a fresh mixture of HCl and HNO_3_ (1:3, *v*/*v*) was used to digest 0.2 g of soil samples in a water bath for 2 h. Then, the concentrations of As and Hg in the digested solutions were measured by atomic fluorescence spectrophotometry (AFS-9130, Titan Instruments, Beijing, China) [30]. For Cd, Cu, Cr, Mn, Pb, and Zn analysis, 8 mL of mixed acid of HCl and HNO_3_ (1:3, *v*/*v*) were used to digest 0.2 g soil samples at 105 °C for 5 h. Then, the concentrations of the five metals in the digestion solutions were determined by inductively coupled plasma mass spectrometry (ICP-MS 7500x, Agilent Technologies, Santa Clara, CA, USA) [31]. To ensure precision and reproducibility of analytical results, all the analyses were performed on duplicate subsamples, with blanks and standard reference materials (GBW07309, Chinese Academy of Measurement Sciences, Beijing, China) added for each batch of samples (1 blank and 1 standard for every 10 samples). The recoveries of samples spiked with standards ranging from 92 to 106%. The soil and sediment organic matter (SOM) was measured by wet combustion, and pH in a 1:5 soil: water solution.

### 2.3. Geo-Accumulation Index

The *I_geo_* was introduced by Muller [32]. It can be used to determine whether soils and sediments have been contaminated by metals [33], more generally as a measure of soil and sediment quality [34]. The *I_geo_* for the soils and sediments was calculated using the following equation:

(1) $$Igeo=\log_{2}(C_{n}/1.5\;B_{n}),$$

where *C_n_* is the measured concentration of each metal that was identified in the soils or sediments (mg kg^−1^), and *B_n_* is the geochemical background value that was determined from the concentrations of metals in the A soil layer (0–20 cm) that are naturally present in Jiangsu province, China [35]. The factor of 1.5 is used as a correction for potential variation in the background differences. According to the *I_geo_* value, contamination can be classified in seven grades as shown in Table 1.

### 2.4. Potential Ecological Risk Index

The potential ecological risk index (*RI*) was introduced by Hakanson [36] to assess the degree of toxic metal pollution in soils and sediments. It can also be applied to infer the degree of biological risk of contamination [37,38]. This index was calculated as:

(2) $$RI=\sum_{i=1}^{n}E_{r}^{i}=\sum_{i=1}^{n}\left(T_{r}^{i}\times C^{i}/C_{n}^{i}\right)$$

where *RI* is computed as the sum of all the potential ecological risk indices for individual metals in soils or sediments. *E_r_^i^* is the potential ecological risk index of individual metal *i* in the soil or sediment. *T_r_^i^* is the toxicity response factor for metal *i*, where *T_r_^i^* is 10 for As, 30 for Cd, 2 for Cr, 5 for Cu, 40 for Hg, 1 for Mn, 5 for Pb, and 1 for Zn. *C^i^* is the measured concentration of metal *i* and *C_n_^i^* is the reference value of heavy mental *i* that was collected from the natural geochemical background concentrations of metals at the A soil layer (0–20 cm) in Jiangsu province, China [35]. According to the value of *E_r_^i^* and *RI*, 5 grades of *E_r_^i^* and 4 grades of *RI* can be determined as shown in Table 2.

### 2.5. Statistical Analysis

General calculations and data handling were performed with Excel 2016 (Microsoft, Redmond, WA, USA) and Origin 2021 (Origin Lab, Northampton, MA, USA). Statistical analyses, including analysis of variance (ANOVA), principal component analysis (PCA), and correlation analysis (CA), were performed using SPSS Statistics 25.0 (IBM, Armonk, NY, USA). A one-way ANOVA was used to determine whether there were any statistically significant differences among the five types of land use for the measured parameters in soils and corresponding sediments. PCA and CA were performed to identify the possible sources of metal pollution.

## 3. Results and Discussion

### 3.1. Concentrations of Metals in Surface Soils and Their Corresponding River Sediments under Different Types of Land Use

The metal concentrations, soil organic matter (SOM) content, and pH in the surface soil and its corresponding river sediment under five types of land use are summarized in Table 3. The mean concentration of As in the surface soils that were calculated across all five land use types was 4.0 times higher than the background values for soils in Jiangsu Province (BVSJ), the mean Cd concentration was 1.6 times higher than the BVSJ, the mean Zn concentration was 1.2 times higher than the BVSJ, the mean Cu concentration was 1.1 times higher than the BVSJ, while the mean concentrations of Cr, Hg, Mn, and Pb were lower than their BVSJ [35]. This finding demonstrated that As concentrations were significantly greater than the BVSJ values in all five land use types, indicating that the study area was polluted with As. A comparison of the different land use types revealed that the concentrations of As, Cd, Cr, Cu, Pb, and Zn in the surface soils were significantly higher in Farmland (FL*_so_*) compared to Reed land (RL*_so_*) and Poplar Forest (PF*_so_*) (*p* < 0.05). It could be attributed to the use of fertilizers and pesticides on farmland, both of which can contain metals [39]. According to our field investigation in the study area, the rice-wheat rotation is the most common cropping system on farmland, and the livestock manures, organic, and chemical fertilizers were used extensively to boost crop yields. Annual fertilizer application on agriculture in Jiangsu Province might result in annual inputs of 0.11 g Cd, 6.12 g Pb, 9.63 g Cr, and 20.17 g Zn per hectare [40].

The mean concentration of As in sediments that was calculated across all five land use types was 1.7 times higher than the BVSJ, the mean Cd concentration was 1.4 times higher than the BVSJ, the mean Zn concentration was 1.1 times higher than the BVSJ, the mean Mn concentration was the same as the BVSJ, while the mean concentrations of Cr, Cu, Hg, and Pb were lower than their BVSJ (Table 3). This finding is in agreement with a previous study that showed Cd and As were the predominant contaminants in the sediments of Hongze Lake [27]. Further statistical analysis revealed that there were no significant differences in the sediment Cd and Hg concentrations among FL*_sd_*, RL*_sd_*, PF*_sd_*, and WF*_sd_* (*p* > 0.05, Table 3). However, the concentrations of Cd and Hg in the sediments under Culture Ponds (CP*_sd_*) were significantly higher than those that were under other types of land use (*p* < 0.05). This result may be explained by the fact that large amounts of exogenous aquatic feed release Cd, Zn, Cu, Cr, and other metal elements into the water body, and then accumulate in the sediments of aquaculture ponds [41]. It is interesting to note that the concentrations of Cd and Hg in sediments declined along the direction of water flow, from CP*_sd_* to WF*_sd_*, indicating a potential pollution risk of these metals in the river sediments that were associated with culture ponds. Besides, the mean concentrations of As, Cr, Cu, Mn, Pb, and Zn in FL*_sd_* were all significantly higher than those under other types of land use (*p* < 0.05). It may be related to anthropogenic processes such as metal inputs from fertilizers and organic manures, irrigation, and atmospheric deposition into farmland. With agricultural runoff transporting those metals into the watercourse through water exchange in the rice fields, they eventually accumulate in their corresponding river sediments [42,43].

The soil and sediment physicochemical properties such as the pH and SOM can affect the accumulation of metals [44,45]. As shown in Table 3, the pH values in the CP*_so_* were significantly lower than those in the RL*_so_* (*p* < 0.05), and slightly lower than those in the FL*_so_*, PF*_so_*, and WF*_so_* (*p* > 0.05). The relatively low pH in the CP*_so_* could be associated with the adjustment of pH in this system to meet aquaculture requirements [46]. The contents of SOM in FL*_so_* were significantly greater than those in other types of land use soils. One possible explanation is the region’s heavy use of organic fertilizers, which may result in soil organic matter accumulation [47]. Similarly, the contents of SOM in the FL*_sd_* were significantly higher than those in CP*_sd_* (*p* < 0.05) and slightly higher than those in other types of land use-corresponding sediments. It may be due to the offsite movement of fertilizers that were used in farmland sites to the adjacent sediment samples [48].

### 3.2. Metal Pollution in Surface Soil and Sediment

**Figure 2:**
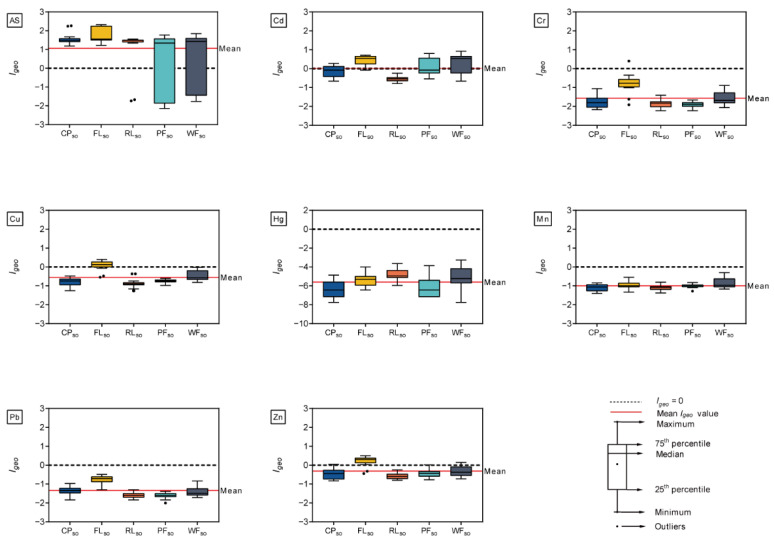
Box-plot of geo-accumulation index (*I_geo_*) of metals in the surface soil that were collected from CP*_so_*, FL*_so_*, RL*_so_*, PF*_so_*, and WF*_so_*. The red solid line represents the mean *I_geo_* values that were averaged across all types of land use, and the black dashed line represents *I_geo_* = 0.

The geo-accumulation index (*I_geo_*) can be used to determine whether soils and sediments have been contaminated by metals [33], more generally as a measure of soil and sediment quality [34]. According to the pollution classification [32], the mean *I_geo_* values for Cr, Cu, Hg, Mn, Pb, and Zn elements under all types of land use belonged to class zero, indicating that the surface soils in the study area were not polluted by these metals (Figure 2). However, the surface soils from all types of land use were moderately polluted by As (*I_geo_* = 1.06) but ranged from being unpolluted to moderately polluted by Cd (*I_geo_* = 0.02). There were significant differences among the five types of land use. The FL*_so_*, CP*_so_*, and RL*_so_* soils were moderately contaminated with As, whereas the WF*_so_* and PF*_so_* soils were uncontaminated to moderately contaminated with As and Cd. Furthermore, the FL*_so_* soils were uncontaminated to moderately contaminated by Cd, Cu, and Zn. As mentioned before, substantial anthropogenic activities such as the use of organic and chemical fertilizers, livestock manures, and aquatic feed under FL*_so_* and CP*_so_* could account for the greater pollution of metals under these two types of land use [49].

**Figure 3:**
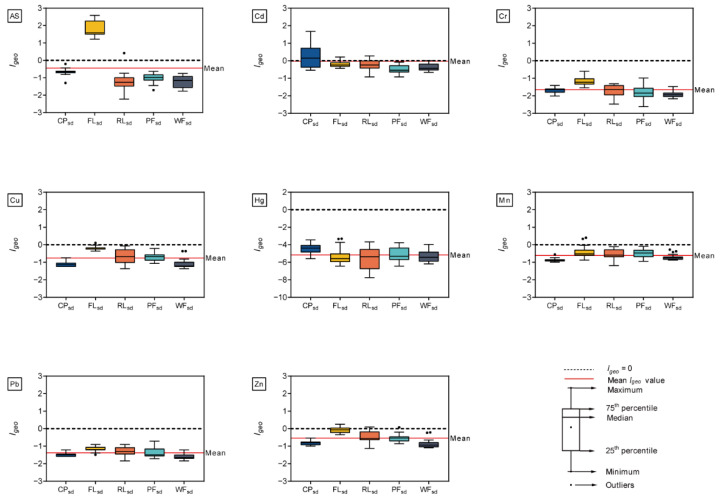
Box-plot of geo-accumulation index (*I_geo_*) of metals in the surface soil that were collected from CP*_sd,_* FL*_sd_*, RL*_sd_*, PF*_sd_*, and WF*_sd_*. The red solid line represents the mean *I_geo_* values that were averaged across all types of land use, and the black dashed line represents *I_geo_* = 0.

As shown in Figure 3, the mean *I_geo_* values that were averaged across all types of land use were −0.45 for As, −0.21 for Cd, −1.65 for Cr, −0.75 for Cu, −5.61 for Hg, −0.61 for Mn, −1.38 for Pb, and −0.54 for Zn in the sediments, placing them into the class of practically unpolluted. However, the level of metal pollution in sediments varied among the five land use types. Specifically, the sediments from CP*_sd_* were uncontaminated to moderately contaminated with Cd (*I_geo_* = 1.78), whereas FL*_sd_* sediments were moderately contaminated with As (*I_geo_* = 0.31). These results are consistent with those of previous studies [50] and suggest that anthropogenic activities such as fertilization on agricultural fields and baiting in aquaculture ponds might pollute the sediments of nearby wetlands.

### 3.3. Potential Ecological Risk of Metals

**Figure 4:**
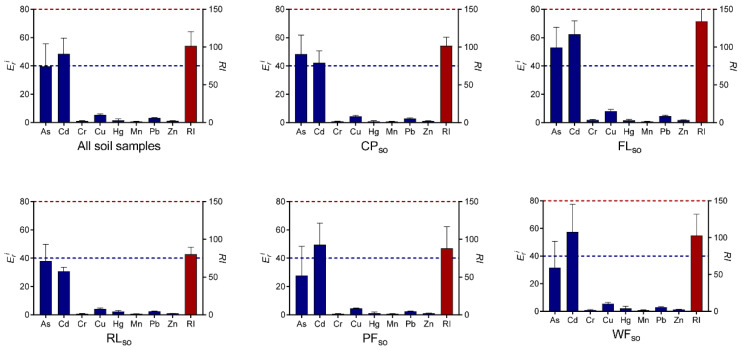
The potential ecological risk index (*RI*, *E_r_^i^*) of metals in the surface soil samples that were associated with five types of land use (CP, FL, RL, PF, and WF). The blue bar-plot on the left Y-axis represents the mean *E_r_^i^* values of the 10 or 50 replicate samples per metal, and the blue dashed line indicates *E_r_^i^* = 40. The red bar-plot on the right Y-axis represents the mean *RI* value of the 10 or 50 replicate samples, and the red dashed line indicates *RI* = 150.

The potential ecological risk index can be used to assess the degree of toxic metal pollution in soils and sediments [36]. It can also be applied to infer the degree of biological risk of contamination [37,38]. The potential ecological risk index of individual metal (*E_r_^i^*) and all metals (*RI*) are shown in Figure 4. The mean *E_r_^i^* values for all the surface soils were in the following order: Cd > As > Cu > Pb > Hg > Zn > Cr > Mn. This means that the Cd and As concentrations posed a greater risk to the environment than the other elements. In particular, the potential ecological risk index for Cd (*E_r_^i^* = 48.47) exhibited moderate pollution. However, the mean *E_r_^i^* values of Pb, Hg, Zn, Cr, and Mn were lower than 40, which suggests slight potential ecological risk of the corresponding metals in all the surface soils. In addition, the *RI* values for all the surface soils across five land use types ranged from 45 to 167, with a mean of 101. This indicates that the measured metals in surface soils pose a low overall ecological risk. The mean *RI* values decreased in the following order for the five types of land use: FL*_so_* > WF*_so_* > CP*_so_* > PF*_so_* > RL*_so_*. This finding implies that farming poses the largest potential ecological risk of the five land use types that were studied. The mean *E_r_^i^* values of As and Cd in farmland soils were greater than 40, indicating that these two metals were the primary elements that pose a potential ecological risk to the environment.

**Figure 5:**
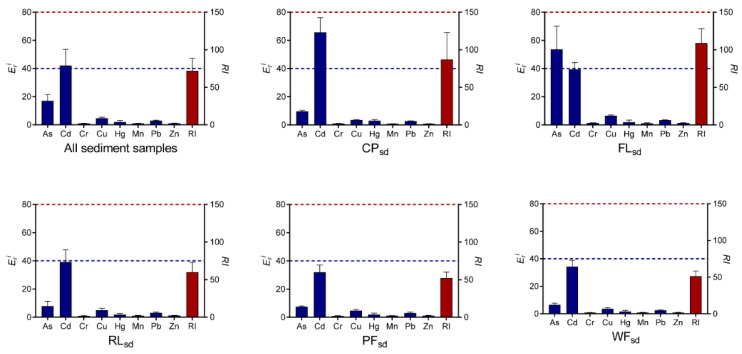
The potential ecological risk index (*RI*, *E_r_^i^*) of metals in sediment samples that were associated with five types of land use (CP, FL, RL, PF, and WF). The blue bar-plot on the left Y-axis represents the mean *E_r_^i^* values of the 10 or 50 replicate samples per metal, and the blue dashed line indicates *E_r_^i^* = 40. The red bar-plot on the right Y-axis represents the mean *RI* value of the 10 or 50 replicate samples, and the red dashed line indicates *RI* = 150.

Figure 5 depicts the potential ecological risk index (*RI*, *E_r_^i^*) of metals in sediment samples that were associated with five different types of land use. The mean *E_r_^i^* values for individual metals that were averaged across five types of land use were ranked as Cd > As > Cu > Pb > Hg > Zn > Mn > Cr, indicating the concentrations of Cd had greater potential ecological risk than other elements in the sediments. The *RI* values for all the sediment samples ranged from 42 to 168, with a mean of 71. This result indicates that the metals that were measured in this study pose a low overall ecological risk. For the five types of land use, the mean values of *RI* decreased in the order of FL*_sd_* > CP*_sd_* > RL*_sd_* > PF*_sd_* > WF*_sd_*. This suggests that the sediments from the FL*_sd_* and CP*_sd_* land use types posed a higher potential ecological risk than the sediments from other types of land use. The mean *E_r_^i^* values of Cd (*E_r_^i^* = 65.68) in CP*_sd_* and As (*E_r_^i^* = 53.65) in FL*_sd_*, posed a moderate risk to the environment. Furthermore, the present study further supported the previous observations that Cd and As posed the higher potential ecological risk for the Hongze Lake wetland [27,29,50].

**Figure 6:**
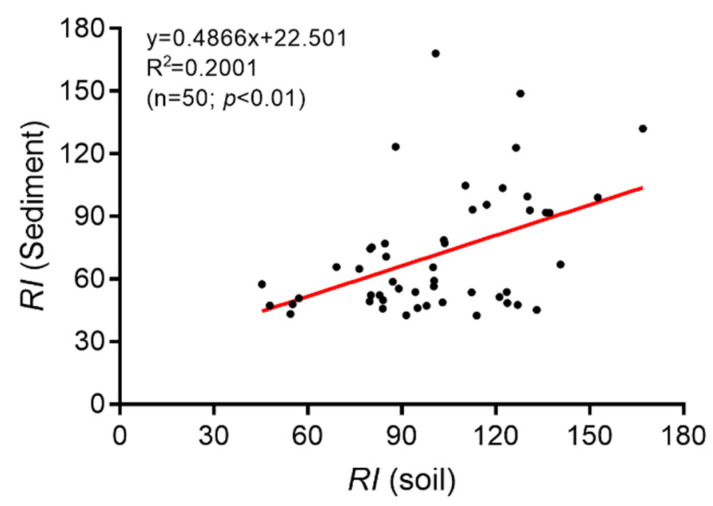
The linear relationship of RI between the soils and its corresponding river sediments.

This study demonstrated that the concentrations of metals in the sediments were closely related to those in the adjacent soils. There was a significant and positive correlation between the soil *RI* values and their corresponding sediment *RI* values (Figure 6). This indicates that the risk of pollution in wetland sediments increased with the increasing risk of pollution in the adjacent soils. The present finding is in line with previous research, which found that land use activities near wetlands had a significant impact on the sediment metal concentrations [51].

### 3.4. Identification of Possible Sources of Metals

**Figure 7:**
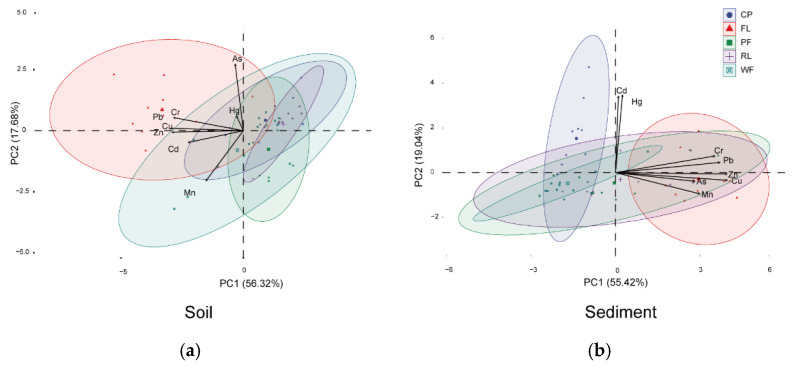
Results of principle component analysis of metal concentrations in the surface soil (**a**) and sediment (**b**) samples.

**Figure 8:**
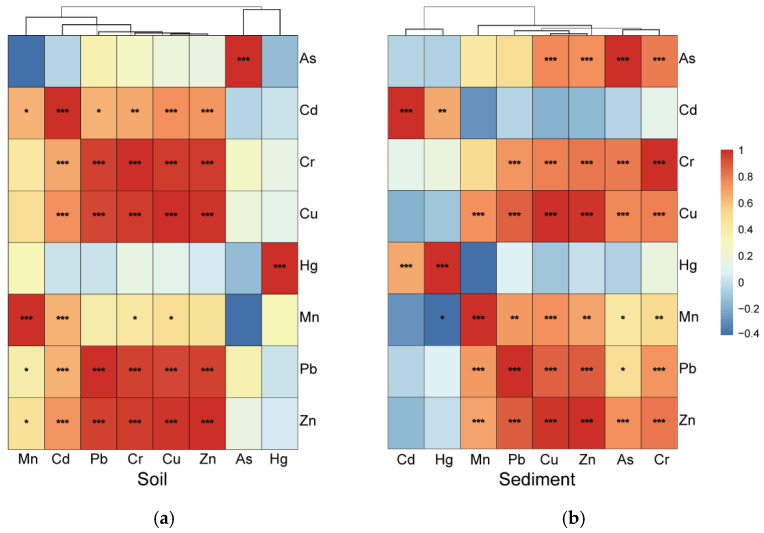
Correlations of metals in the surface soil (**a**) and sediment (**b**) samples. The colors red and blue represent positive and negative correlations, respectively. * Significant correction at the level of *p* < 0.05; ** Significant correction at the level of *p* < 0.01; *** Significant correction at the level of *p* < 0.001.

Various multivariate approaches have previously proved effective for source identification of metals in environmental samples [52,53,54]. In this study, both PCA and CA were used to identify the possible source of metals in the soils and sediments. The results are shown in Figure 7 and Figure 8. For the surface soils, the first two principal components (PC1 and PC2) explained 73.99% of the total variance in the soil metals. The PC1, explaining 56.32% of the total variance, was strongly and positively related to the concentrations of Cd, Cr, Cu, Pb, and Zn (Figure 7a), which were strongly correlated (Figure 8a). It suggests that Cd, Cr, Cu, Pb, and Zn may come from the same source. The concentrations of Cd, Cr, Cu, Pb, and Zn in soils varied among the different types of land use, with the highest level in FL*_sd_* and lowest in RL*_sd_* (Table 3). This suggests that anthropogenic activities had an impact on the spatial distribution of Cd, Cr, Cu, Mn, Pb, and Zn in surface soils. The application of chemical and organic fertilizers and livestock manure in the study area are likely an important source of Cd, Cr, Cu, Pb, and Zn pollution [28,55,56,57]. The previous study has reported concentrations of Cd in chemical fertilizers, Pb and Cr in commercial organic fertilizers, and Cu and Zn in livestock manure were relatively high in this area [49]. In addition, the application of local residential waste onto farmland may provide another origin of Zn, Cu, and Cd in topsoil [58]. The PC2, explaining 17.68% of the total variance, exhibited strong loadings of As and Mn concentrations. Many previous studies have reported that a high concentration of As may be associated with coal combustion and industrial production followed by atmospheric deposition [57,59]. However, according to our field investigation, the study area has a history of long-term irrigation with treated sewage from the Laozishan town domestic sewage treatment plant, which could be the most likely source of As pollution. Although Mn is normally derived from geogenic sources [53,60], it was grouped into PC2 with a factor loading of >0.5 in this study. This finding implies that Mn was primarily related to geological anomalies or ore mining [61]. Moreover, the weak correlation between As and Mn in the surface soil (Figure 8a) further ruled out the possibility of agricultural and atmospheric sources as the sole source of contamination.

For the wetland sediments, the first two principal components (PC1 and PC2) explained 74.46% of the total variance in sediment metals. The elements with high positive factor loading on PC1 (Figure 7b) were As, Cr, Cu, Mn, Pb, and Zn, and the Pearson correlation coefficients among these metals ranged from 0.43 to 0.98 (*p* < 0.05) (Figure 8b), indicating similar origins. The concentrations of As, Cr, Cu, Mn, Pb, and Zn in the sediments varied among the different types of land use, with higher levels in CP*_sd_* or FL*_sd_* and lower levels in WF*_sd_* (Table 3). It further confirmed that the adjacent land use had an impact on the spatial distribution of these metals. PC2, explaining 19.04% of the total variance, exhibited highly positive factor loadings for both Cd and Hg. The concentrations of these two metals were highly correlated across the samples (*p* < 0.01; Figure 8b), indicating a shared source which significantly different from the source of other heavy elements. As discussed before, polluted water from industrial activities was discharged into the Hongze Lake from the Huai River, and inevitably contributed to the relatively high pollution levels in the sediments. The discharge of metal-containing wastewater from metallurgical plants, chemical plants, coking plants, electroplating plants, and leather factories is the main source of Cd and Hg in the Huai River, which causes the Cd content in the river to rise dramatically, with maximum and average values of 6.86 and 2.07 times higher than the average value (0.14 mg/kg) of Cd content in China water system sediments [50].

## 4. Conclusions

This study demonstrated that the land use activities in the study area had a large impact on the metal pollution in the sediments of lake wetlands. Farmlands and culture ponds had a significantly greater impact on the metal concentrations of their surrounding sediments than reed land, poplar forests, and willow forests. The long-term accumulation of metals from these two land use practices also posed a greater ecological threat to the river sediments around them. Furthermore, this study revealed that the pollution risk of metals in wetland sediments increased with the increasing risk of metal pollution in the adjacent soils. The results of multivariate analysis further confirmed that the adjacent land use had an important impact on the sources and spatial distribution of these metals. Our findings have important implications for the development of pollution prevention and control strategies to reduce metal pollution for the wetlands of this region.

## Figures and Tables

**Table 1:** Pollution classification of geo-accumulation index.

*I_geo_*	≤0	0–1	1–2	2–3	3–4	4–5	≥5
Pollution degree	Practically unpolluted	Unpolluted to moderately polluted	Moderately polluted	Moderately to heavily polluted	Heavily polluted	Heavily to extremely polluted	Extremely polluted
Levels	0	1	2	3	4	5	6

**Table 2:** Ecological risk classification of metals.

Assessment Criterion	Low Risk	Moderate Risk	High Risk	Very High Risk	Extremely High Risk
*E_r_^i^*	≤40	40 < *E_r_^i^* ≤ 80	80 < *E_r_^i^* ≤ 160	160 < *E_r_^i^* ≤ 320	>320
*RI*	≤150	150 <*RI* ≤ 300	300 < *RI* ≤ 600	*RI* > 600	

**Table 3:** Metal concentrations together with the pH and SOM in surface soils and its corresponding sediments under different types of land use.

	Land Uses	pH	SOM (g/kg)	As (mg/kg)	Cd (mg/kg)	Cr (mg/kg)	Cu (mg/kg)	Hg (mg/kg)	Mn (mg/kg)	Pb (mg/kg)	Zn (mg/kg)
Soil	CP*_so_*	8.03 ± 0.15 ^b^	3.84 ± 0.96 ^b^	48.40 ± 13.97 *^a^	0.18 ± 0.14 ^c^	35.55 ± 8.26 ^bc^	19.65 ± 2.87 *^c^	0.006 ± 0.005 *^b^	418.85 ± 53.31 *^b^	15.40 ± 2.26 *^b^	70.30 ± 13.53 *^bc^
	FL*_so_*	8.09 ± 0.26 ^ab^	10.35 ± 1.69 *^a^	53.00 ± 14.97 ^a^	0.26 ± 0.04 *^a^	71.25 ± 24.51 *^a^	36.20 ± 5.69 *^a^	0.012 ± 0.006 ^ab^	453.20 ± 65.00 *^b^	23.65 ± 3.38 *^a^	110.95 ± 16.97 *^a^
	RL*_so_*	8.39 ± 0.21 *^a^	1.77 ± 0.25 *^c^	38.01 ± 11.54 *^b^	0.13 ± 0.01 *^d^	32.80 ± 4.89 ^bc^	18.55 ± 2.95 ^c^	0.017 ± 0.008 ^a^	413.85 ± 39.74 *^b^	13.20 ± 1.24 *^c^	63.30 ± 7.63 ^c^
	PF*_so_*	8.25 ± 0.25 ^ab^	2.95 ± 0.46 ^bc^	27.64 ± 20.20 *^b^	0.21 ± 0.07 *^bc^	31.50 ± 3.22 ^c^	20.20 ± 1.28 ^c^	0.008 ± 0.006 *^b^	441.55 ± 31.12 *^b^	12.94 ± 1.17 *^c^	69.55 ± 9.31 ^bc^
	WF*_so_*	8.28 ± 0.20 ^ab^	3.16 ± 1.31 ^bc^	31.73 ± 18.56 *^b^	0.24 ± 0.08 *^ab^	40.80 ± 10.56 *^b^	24.45 ± 4.48 *^b^	0.016 ± 0.011 ^a^	493.30 ± 92.81 ^a^	15.20 ± 2.84 *^b^	76.15 ± 14.75 *^b^
Sediment	CP*_sd_*	8.07 ± 0.36 ^b^	2.60 ± 1.12 ^b^	9.62 ± 1.85 *^b^	0.28 ± 0.20 ^a^	36.72 ± 3.74 ^b^	15.55 ± 1.72 *^c^	0.021 ± 0.008 *^a^	480.97 ± 35.48 *^b^	14.11 ± 1.11 *^b^	53.56 ± 4.57 *^c^
	FL*_sd_*	8.30 ± 0.08 ^ab^	5.07 ± 0.34 *^a^	53.65 ± 16.39 ^a^	0.17 ± 0.02 *^b^	52.30 ± 8.57 *^a^	29.20 ± 2.48 *^a^	0.014 ± 0.012 ^b^	670.90 ± 182.75 *^a^	17.45 ± 1.67 *^a^	89.55 ± 10.83 *^a^
	RL*_sd_*	8.06 ± 0.05 *^b^	4.93 ± 1.40 *^a^	7.90 ± 4.66 *^b^	0.16 ± 0.04 *^b^	36.25 ± 8.28 ^bc^	21.95 ± 6.20 ^b^	0.013 ± 0.010 ^b^	617.25 ± 117.25 *^a^	16.30 ± 2.77 *^a^	70.70 ± 15.32 ^b^
	PF*_sd_*	8.27 ± 0.13 ^ab^	4.57 ± 1.60 ^a^	7.52 ± 1.24 *^b^	0.13 ± 0.02 *^b^	35.30 ± 11.23 ^bc^	21.10 ± 3.70 ^b^	0.014 ± 0.008 *^b^	632.70 ± 103.55 *^a^	15.90 ± 3.55 *^a^	67.65 ± 12.05 ^b^
	WF*_sd_*	8.37 ± 0.07 ^a^	3.64 ± 0.78 ^ab^	6.54 ± 1.49 *^b^	0.14 ± 0.02 *^b^	31.40 ± 4.17 *^c^	16.05 ± 3.76 *^c^	0.012 ± 0.006 ^b^	542.00 ± 70.40 ^a^	13.10 ± 1.68 *^b^	52.55 ± 10.57 *^c^
BVSJ	Soils	-	-	10.00	0.13	77.80	22.30	0.29	585.00	26.20	62.60

Data are shown as the mean ± SD. abcd Values in each column with the same letter are not significantly different between different sites (*p* < 0.05). * Represent significantly different (*p* < 0.05) between soils and sediments. BVSJ—Background values for soils in Jiangsu province.

## Data Availability

Not applicable.
